# Comparative analysis of precursor supply and pathway reinforcement strategies for shikimic acid biosynthesis in *Pseudomonas putida*

**DOI:** 10.1186/s12934-026-03042-6

**Published:** 2026-07-22

**Authors:** İrem İlter, Tanya Beril Korkmaz, Hatice Arslan, Ahmet Kalaycı, Emrah Kırdök, Tomás Aparicio, Özlem Akkaya

**Affiliations:** 1 Department of Molecular Biology and Genetics, Faculty of Basic Sciences, Gebze Technical University, 41400 Kocaeli, Turkey; 2 Department of Biotechnology, Faculty of Science, Mersin University, Mersin, Turkey; 3 Department of Medical Biology, Faculty of Medicine, İstinye University, Istanbul, Turkey; 4 Systems Biology Program, Centro Nacional de Biotecnología (CNB-CSIC), Madrid, Spain; 5 Central Research Laboratory Application and Research Center (GTUMAR), Gebze, Kocaeli Turkey

**Keywords:** Shikimic acid, *Pseudomonas putida*, Synthetic biology, Metabolic engineering, Precursor rerouting, Comparative transcriptomics

## Abstract

The shikimate pathway is a central metabolic route for the biosynthesis of high-value aromatic compounds. Traditional metabolic engineering strategies have largely focused on overexpressing pathway-specific genes, enhancing precursor supply, or introducing feedback-resistant variants of 3-deoxy-D-arabino-heptulosonate 7-phosphate (DAHP) synthase to increase pathway flux. However, how these distinct strategies differentially affect flux distribution and the accumulation of key intermediates, specifically 3-dehydroshikimate (DHS) and shikimic acid (SA), remains largely unexplored in *Pseudomonas putida* (*P. putida*). Here, we systematically assess the effects of commonly applied engineering strategies on DHS and SA production using distinct SA pathway modules. The results show that overexpression of transketolase I (*tktA*), phosphoenolpyruvate synthase (*ppsA*), and SA pathway genes (*aroE*, *aroB*, *aroQ-II*), together with heterologous expression of feedback-resistant *aroG*, in a genome-edited *P. putida* EM42 strain harboring *pykA*/*pykF* deletions and a *hexR* knockout, yielded approximately 71 mg L⁻^1^ shikimic acid, corresponding to a > 284-fold increase relative to the wild-type EM42. Notably, the findings reveal that following the relief of precursor constraints, the achievable maximum titer is unaffected by the particular DAHP synthase isoform utilized. Furthermore, the standalone overexpression of *ppsA* and *tktA* yielded SA and DHS levels comparable to those achieved with the additional expression of the aforementioned pathway genes. However, despite this phenotypic convergence in titers, comparative transcriptomic analysis revealed that the genetic route taken to reach these levels is associated with markedly distinct transcriptional configurations of the host metabolic network. In the same genetic background, amplification of *ppsA*-*tktA* alone is associated with an increased transcriptional investment in the biosynthesis of essential cofactors and metabolic precursors, whereas overexpression of the SA pathway is accompanied by enrichment of protocatechuate catabolic genes, consistent with the intrinsic aromatic funneling capacity of *P. putida* under conditions of elevated shikimate pathway flux. These results demonstrate that evaluating engineering success solely based on final product yields obscures substantial differences in underlying metabolic states. Accordingly, we propose that tailored engineering strategies guided by key pathway intermediates are critical for the rational optimization of *P. putida* as an efficient cell factory.

**Supplementary Information:**

The online version contains supplementary material available at https://doi.org/10.1186/s12934-026-03042-6.

## Introduction

Aromatic compounds and their functionalized derivatives are indispensable to the pharmaceutical, nutraceutical, and polymer industries. In biological systems, the biosynthesis of these molecules is predominantly governed by the shikimate pathway, a central metabolic route that directs carbon flux toward aromatic amino acids and a myriad of specialized metabolites. Beyond its physiological role, this pathway serves as a primary biosynthetic platform for sustainable manufacturing. By leveraging its inherent flexibility, microbial cell factories can be engineered to produce value-added commodities ranging from cis,cis-muconic acid and tryptophan to salicylic acid, vanillin, and 2-phenylethanol [1–5]. Consequently, this route represents a strategic target for metabolic engineering to facilitate the transition toward bio-based production [1, 4, 6]. Shikimic acid (SA; 3,4,5-trihydroxycyclohex-1-carboxylic acid) is a key intermediate in this pathway, functioning as a central precursor for the synthesis of various downstream aromatic metabolites [1, 4, 7, 8]. SA is also the starting material for the industrial production of the antiviral neuraminidase inhibitor oseltamivir phosphate (Tamiflu®) [6, 9, 10]. To date, diverse microbial production platforms including *Escherichia coli* (*E. coli*), *Bacillus* spp., *P. putida*, and *Corynebacterium glutamicum* (*C. glutamicum*) have been strategically engineered for the biosynthesis of shikimic acid (SA) and its value-added derivatives [1, 10–13].

Owing to its genetic tractability and robust metabolic capacity, *P. putida* KT2440 has emerged as a prominent chassis for synthetic biology. Its high tolerance to aromatic-induced toxicity, combined with an extensive catabolic repertoire, makes it an ideal host for the valorization of complex substrates under harsh industrial conditions [14–16]. These attributes, supported by well-characterized pathways, enable the efficient conversion of diverse feedstocks into commercially relevant chemicals [17, 18]. Moreover, *P. putida* exhibits a sophisticated sugar metabolism that deviates from the canonical model; it lacks a functional Embden-Meyerhof-Parnas (EMP) glycolysis and instead channels glucose through convergent peripheral pathways into 6-phosphogluconate, which subsequently feeds the Entner–Doudoroff (ED) pathway. This metabolic arrangement, integrated with the gluconeogenic EMP and pentose phosphate (PP) routes, constitutes the EDEMP cycle—a unique architecture that ensures balanced carbon distribution and robust cofactor supply [19]. In this organism, glucose can be phosphorylated internally by glucokinase (*glk*) or oxidized in the periplasm to gluconate (and 2-ketogluconate) by glucose dehydrogenase (*gcd*) [20]. Gluconate is further phosphorylated by gluconate kinase, or converted to 6-phosphogluconate via a dedicated 2-ketogluconate route. The regulation of glucose uptake and the downstream catabolism of sugars is orchestrated, among other factors, by the two-component system regulator GltR-II (PP_1012) as well as the transcriptional repressor HexR (PP_1021) [20–24]. All three routes converge at 6-phosphogluconate, which is metabolized by ED enzymes 6PG dehydratase (*edd*) and KDPG aldolase (*eda*) to yield pyruvate and glyceraldehyde-3-phosphate. This ED-centric metabolism provides a high NADPH yield per glucose consumed [25, 26], offering a clear advantage for enhancing flux through the shikimate pathway, since NADPH is required for the conversion of DHS to SA catalyzed by the shikimate dehydrogenase (*aroE*) [27–29].

The shikimate pathway begins with the condensation of phosphoenolpyruvate (PEP) and erythrose-4-phosphate (E4P) into 3-deoxy-D-arabino-heptulosonate 7-phosphate (DAHP). This rate-limiting step, mediated by DAHP synthase, acts as a metabolic gatekeeper subject to stringent feedback inhibition by terminal aromatic amino acids. To bypass these regulatory constraints, a prevalent engineering strategy involves the deployment of feedback-inhibition-resistant (fbr) enzyme variants to sustain carbon flux [30–32]. While *P. putida* possesses three putative DAHP synthase isoenzymes (aroF-I, aroF-II, and aroH), their specific physiological contributions remain to be fully elucidated [33]. Following DAHP formation, a series of conserved enzymatic steps lead to the production of shikimate and subsequently chorismate. As the definitive branch point, chorismate directs flux toward the biosynthesis of phenylalanine, tyrosine, and tryptophan, alongside diverse value-added aromatic metabolites [8, 34, 35]. Consequently, intermediates like DHS and chorismate serve as critical nodes for metabolic redirection toward high-value industrial products [36].

In *E. coli*, strategies to enhance carbon flux toward the shikimate pathway have primarily focused on increasing the availability of key precursors, PEP and E4P. This is typically achieved by deleting pyruvate kinase genes (*pykA* and *pykF*) to reduce PEP consumption [37–41] or by overexpressing *ppsA* and *tktA* to boost precursor pools [29, 42–45]. Beyond precursor supply, a widely adopted approach involves the overexpression of key pathway enzymes, particularly feedback-resistant DAHP synthases and downstream catalytic steps, to increase overall pathway throughput and promote aromatic biosynthesis [42, 46].

Building on these established frameworks, related strategies have been applied to *P. putida* to produce commercially important derivatives such as p-hydroxybenzoate and cis,cis-muconate [2, 36]. However, the extent to which these modifications differentially influence the partitioning between the two central key intermediates—DHS and SA—remains systematically unexamined. To address this, we utilize *P. putida* EM42, a genome-reduced derivative characterized by enhanced energy and redox status [47–49], to systematically compare established metabolic engineering strategies for DHS and SA production. Notably, even when distinct engineering strategies led to similar metabolite yields, this study further examined the transcriptional landscapes of the selected strains to better understand how these modifications may differentially shape the broader metabolic network.

## Materials and methods

### Bacterial strains and media

The strains used in this study are summarized in Supplementary Table S1. Unless stated otherwise, *P. putida* strains were grown at 30 °C in Luria–Bertani (LB) medium (10 g L^-1^ peptone, 5 g L^-1^ sodium chloride, and 5 g L^-1^ yeast extract) or M9 minimal medium (6 g L^-1^ Na_2_HPO_4_, 3 g L^-1^ KH_2_PO_4_, 1.4 g L^-1^ (NH_4_)_2_Cl_2_, 0.5 g L^-1^ NaCl, 0.2 g L^-1^ MgSO_4_.7H_2_O). *E. coli* was grown at 37 ºC in LB. Following mating procedures, *Pseudomonas* strains were selected on Cetrimide Agar supplemented with appropriate antibiotics. For shikimate production assays, M9 minimal medium containing 20 mM glucose as the sole carbon source was used. When appropriate, antibiotics were added to maintain plasmids: kanamycin (50 µg mL^-1^ for *E. coli* and *P. putida*), ampicillin (50 µg mL^-1^ for *E. coli*; 500 µg mL^-1^ for *P. putida*), or chloramphenicol (30 µg mL^-1^ for *E. coli*). When stated, cultures were induced with IPTG (0.1 to 1 mM) and/or 1 mM 3-methylbenzoate.

### Recombinant DNA techniques

Standard molecular biology protocols were used for DNA manipulation. Primers were purchased as unmodified DNA oligonucleotides from Oligomer (Turkey). Amplification of clonal DNA sequences was carried out using the Q5 High-Fidelity DNA Polymerase (NEB) with primers listed in Table S2. DNA fragments were recovered and purified from agarose gels using the NucleoSpin Gel & PCR Clean-up Kit (Macherey–Nagel, Düren, Germany), following the manufacturer’s instructions. Plasmid assembly was performed using either conventional restriction–ligation techniques or Gibson assembly [50] with the NEB Gibson Assembly® cloning kit (New England Biolabs, Ipswich, MA, USA). Nucleic acid–modifying enzymes were obtained from New England Biolabs. Plasmids were isolated using the Qiagen Miniprep kit. Diagnostic colony PCRs were used to screen *E. coli* and *P. putida* transformants, employing the HOT FIREPol® Blend Master Mix RTL (12.5 mM MgCl_2_) (Solis Biodyne). All plasmid sequences and gene deletions subsequently were verified by MACROGEN Inc. using Sanger sequencing.

### Construction of plasmids

The plasmids used in this study are summarized in Supplementary Table S1 [47–49, 51–58].

### Construction of SA modules featuring distinct DAHP synthase isoforms

For module construction, the *aroB* (PP_5078), *aroQ-II* (PP_2407), and *aroE* (PP_0074) genes were PCR-amplified from *P. putida*, while the *E. coli aroG*^D146N^ variant was sourced from the pS3 plasmid (Table S1). The pSEVA238 plasmid served as the backbone, and the gel-purified DNA fragments were Gibson assembled into the broad-host-range vector pSEVA238 after linearization with *Eco*RI/*Xba*I. In this way, the SA modules were positioned downstream of the 3-methylbenzoate inducible *Pm* promoter. After assembly, plasmids were transformed into *E.coli* CC118, transformants selected on LB medium supplemented with kanamycin, grown at 37 °C and checked by miniprep/restriction and module full sequencing.

### Construction of *ppsA* and *tktA* module for enhanced precursor supply

The coding sequences of *ppsA* (encoding phosphoenolpyruvate synthase) and *tktA* (encoding transketolase) were amplified via PCR from the *P. putida* KT2440 genome using the specific primers detailed in Table S2. The resulting amplicons were assembled into a single ~ 4.3 kb genetic fragment (designated *psta*) through overlap extension PCR, following the method described by Horton et al. [59]. This synthetic operon was subsequently digested with *XbaI* and *SpeI* and ligated into the corresponding restriction sites of the pSEVA424. In the generated construct, pSEVA424_psta, the *psta* cassette was strategically positioned downstream of the IPTG-inducible Ptrc promoter. After assembly, plasmids were transformed into *E.coli* CC118, transformants selected on LB medium supplemented with streptomycin, grown at 37 °C and checked by miniprep/restriction and module full sequencing.

### Plasmid delivery into *P. putida* strains

Plasmid delivery was primarily conducted via tri-parental mating, following the protocol described by Martínez‐García and de Lorenzo [47]. Briefly, donor *E. coli* strains harboring the plasmid, the recipient *P. putida* strain, and the helper strain *E. coli* HB101 (pRK600) were grown overnight in LB medium supplemented with appropriate antibiotics. The cultures were harvested, mixed at a 1:1:1 volumetric ratio, and spotted onto LB solid medium. Following a 24-h incubation at 30 °C, the biomass was resuspended and plated onto Cetrimide Agar medium supplemented with relevant antibiotics to selectively isolate *P. putida* transconjugants. Where indicated, electroporation was used for the introduction of DNA assemblies into *E. coli* or purified plasmids into *P. putida*. Procedures were performed using a GenePulser Xcell system (Bio-Rad, Hercules, CA, USA) equipped with 2 mm gap cuvettes. The electrical parameters were set to 2.5 kV, 200 Ω, and 25 μF. After pulsing, cells were immediately recovered in LB medium before plating on appropriate selective agar.

### Genome editing of *P. putida* EM42

Deletion of *hexR* was performed by recombineering with CRISPR-Cas9 counterselection using the system adapted from Aparicio et al. [57]. Spacer sequences were selected using CRISPOR (http://crispor.org) [60]. After editing, plasmids were cured by counter-selection on no-antibiotic media and confirming loss of Cas9 plasmid by sensitivity to kanamycin. Successful deletions were verified by colony PCR and genome sequencing of the loci, and the resulting strain was named EMΔX (EM42ΔhexR).The previously unpublished strain ΔXAF (EM42ΔhexRΔpykAΔpykF), in which the pyruvate kinase isozyme genes *pykA* and *pykF* were sequentially deleted in the EMΔX background, was used as the chassis in this study.

### Shake-flask cultivation

Engineered *P. putida* strains (and controls) were evaluated in batch flask cultures. Glycerol stock cultures were used to inoculate 5 mL LB pre-cultures (with appropriate antibiotics) and grown overnight. These precultures were used to inoculate main cultures in M9 minimal medium supplemented with 20 mM glucose to an initial OD₆₀₀ of 0.1. Induction of the *Pm* promoter in strains carrying the SA module was initiated by adding 1 mM 3-methylbenzoate once the culture reached an OD_600_ of 0.5. For strains harboring the pSEVA424_psta construct, IPTG was supplemented at final concentrations ranging from 0.1 to 1 mM, as specified. In cases where strains contained both genetic systems, both inducers were added simultaneously at the aforementioned concentrations.

Batch cultivations were performed in 250 mL Erlenmeyer flasks containing 50 mL of culture medium. The flasks were incubated at 30 °C with continuous agitation at 170 rpm for up to 72 h. Throughout the fermentation, cell growth (OD_600_) and pH levels were periodically monitored to assess physiological performance. All experiments were performed in biological triplicate to ensure reproducibility, with data reported as the mean ± standard error of the mean (SEM). To ensure genetic stability, plasmid DNA was re-isolated from the *P. putida* strains at the end of the 72-h fermentation. The integrity of the expression modules (SA1, SA2, SA3 and SA4) was verified through colony PCR and Sanger sequencing. Throughout all cultivation stages, appropriate antibiotic selection pressure was maintained to prevent plasmid loss.

Statistical differences between strains were evaluated via one-way analysis of variance (ANOVA), considering a p-value < 0.05 as statistically significant. Samples were collected at defined time intervals (24, 48, and 72 h) and subjected to high-performance liquid chromatography (HPLC) to quantify product accumulation and glucose consumption.

### Analytical methods

SA and DHS were quantified by HPLC (Shimadzu) using UV detection at 210 nm. Culture samples were centrifuged at 10,000 rpm for 5 min, and the resulting supernatants were filtered through 0.22-µm membranes. A 20-µL aliquot of each sample or standard was injected onto a Coregel 87H3 column maintained at 25 °C. The mobile phase consisted of 0.01 M acetic acid in water (A) and acetonitrile (B), delivered at a flow rate of 0.6 mL min⁻^1^. Calibration curves were generated using authentic shikimic acid (Sigma-Aldrich S5375) and DHS (Sigma 05616), and these external standards were used for quantification.

Glucose was measured on the same HPLC system using an InterSustain NH_2_ column with refractive index detection. Isocratic elution was performed with an 80:20 (v/v) acetonitrile:water mixture at a flow rate of 0.6 mL min⁻^1^. All data were processed with Shimadzu LabSolutions software. Calibration was established using glucose standards ranging from 0.2 to 5.4 g L⁻^1^. Experiments were conducted in triplicate, and results are presented as mean ± standard error. Statistical significance was evaluated using one-way ANOVA with a confidence level of p < 0.05.

### RNA sequencing and transcriptomic analysis

For global transcriptional profiling, RNA-seq analysis was conducted on EMΔXAF/SA4-psta and EMΔXAF-psta in comparison with wild-type EM42. Triplicate cultures of each strain were grown in M9 medium containing 20 mM glucose and harvested at 72 h for total RNA isolation using the RNeasy Kit (Qiagen) and subsequently treated with DNase I to remove residual genomic DNA. The RNA integrity number (RIN) of all samples used for RNA-seq analysis was above 8.0, confirming the suitability of the RNA for transcriptomic analysis. The 72 h sampling point was selected to coincide with peak shikimic acid accumulation. rRNA was depleted (RiboZero kit, Illumina) and libraries were prepared using TruSeq Stranded mRNA Library Prep. Paired-end sequencing (2 × 150 bp) was performed on an Illumina NovaSeq (performed by Macrogen Inc.). Raw reads were quality-trimmed using Trimmomatic with a phred quality score of 20 [61]. Processed reads were mapped to the *P. putida* KT2440 reference genome (RefSeq Accession NC_002947.4) using Bowtie2 [62, 63]. Read counts per gene were obtained with HTSeq-count [64]. Differential expression analysis between engineered (EMΔXAF-psta, EMΔXAF/SA4-psta) and control (EM42) was conducted in DESeq2 (R v4.0) with Benjamini Hochberg correction (padj < 0.05) [65–67]. In addition, differential gene expression analysis was performed to identify metabolic changes between EMΔXAF-psta and EMΔXAF/SA4-psta strains. Genes with padj < 0.05 and Log2 fold change (FC) ≥|2|) were considered significantly different. Heatmaps were generated in R by using *dplyr, scales*, *ggplot2*, *dendextend*, *pheatmap*, *kableExtra*, *ggrepel*, *ggpubr* packages [68–72]. RNA-seq data have been deposited in NCBI Sequence Read Archive (PRJNA1161809).

### Enrichment analyses

To elucidate the metabolic alterations in the engineered strains, we performed KEGG enrichment analyses in both strains EMΔXAF-psta and EMΔXAF/SA4-psta using the *simplifyEnrichment* and *clusterProfiler* packages in R [73–77]. A sensitivity analysis strategy was used to distinguish between global metabolic shifts (|FC|≥ 0) and highly pronounced, specific metabolic responses (|FC|≥ 1). We processed upregulated and downregulated gene sets separately to deconstruct distinct metabolic adjustments (padj < 0.05).

### Validation of RNA-Seq data by RT-qPCR

To validate the differential expression patterns observed via RNA-seq, qRT-PCR was performed on a representative panel of 16 genes using the identical total RNA aliquots from *P. putida* EMΔXAF/SA4-psta and *P. putida* EM42 employed for sequencing. These included shikimate pathway components (*aroE*, *aroB*, *aroQ-II*), precursor-supply genes (*ppsA*, *tktA*), central metabolic loci (*glk*, *mdh*, *gapA*, *gcd*, *zwfA*, *edd*, *eda*), and additional differentially expressed genes (e.g., *phhB*). The concentration and purity of the recovered RNA were assessed using a NanoDrop ND-1000 spectrophotometer (Thermo Fisher Scientific). Subsequently, 1 µg of total RNA was reverse-transcribed into cDNA using the RevertAid First Strand cDNA Synthesis Kit (Thermo Fisher Scientific) according to the manufacturer’s protocol. Target-specific primers (Table S3) were designed using Primer3. The qPCR reactions (15 µL) consisted of 7.5 µL of 2 × SYBR Green Master Mix, 0.3 µM of each forward and reverse primer, and 1 µL of diluted cDNA template. Amplification was performed on an Applied Biosystems StepOnePlus™ Real-Time PCR System using the following thermal profile: an initial denaturation at 95 °C for 10 min, followed by 40 cycles of 95 °C for 15 s, 60 °C for 30 s, and 72 °C for 30 s. To ensure reaction specificity, a melt-curve analysis was performed post-amplification, confirming the presence of a single, specific amplicon for each target. Relative gene expression levels were normalized to the housekeeping gene *rpoD*. Fold-change values were calculated using the 2^–ΔΔCt method, with the EM42 strain serving as the reference control. Statistical significance was determined via one-way ANOVA (p < 0.05).

## Results

### Synergistic enhancement of SA biosynthesis via modular design and chassis optimization

**Fig. 1:**
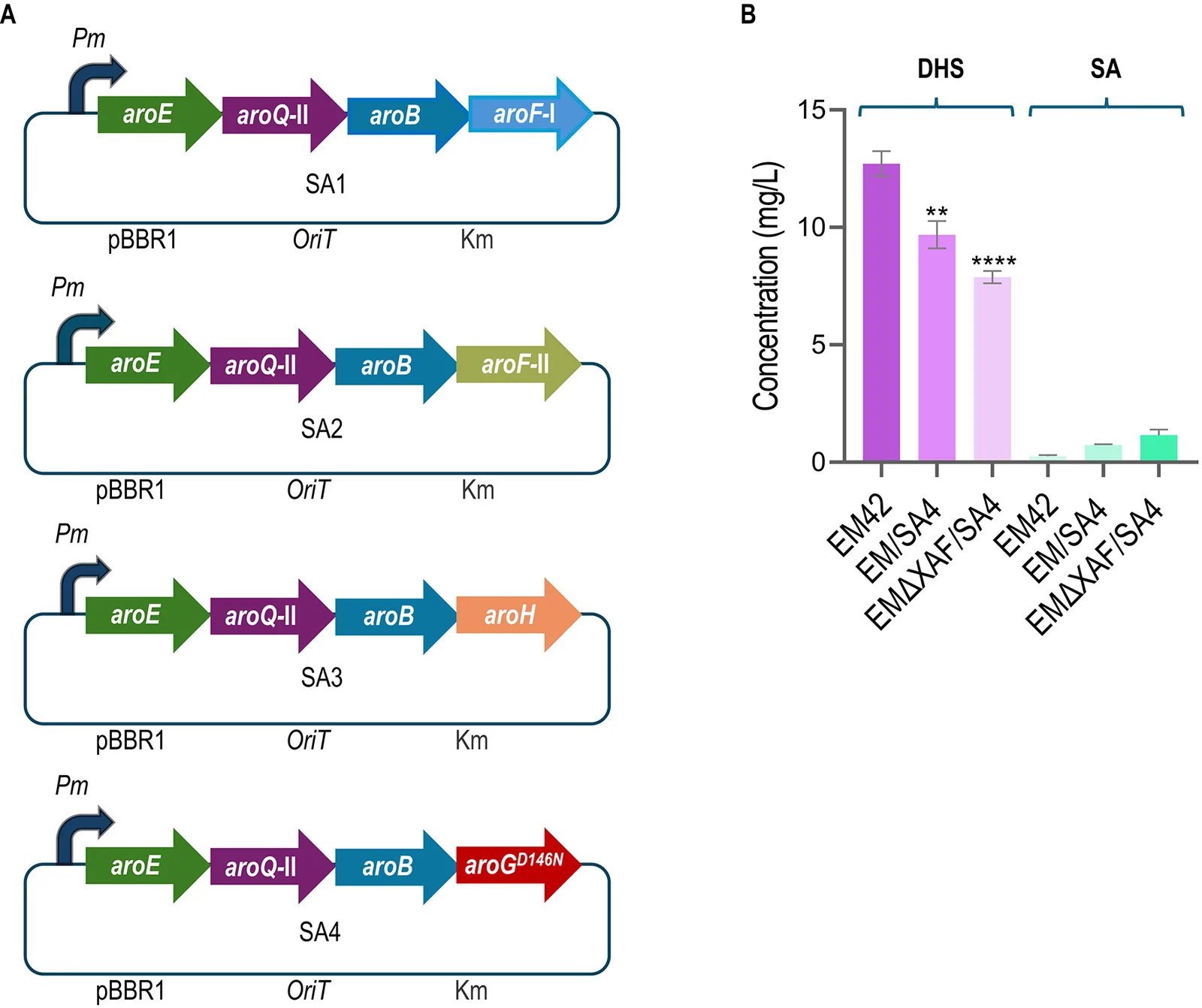
Schematic representation of the SA module variants (SA1–SA4) (not to scale). Each module comprises a polycistronic operon driven by the *Pm* promoter and includes *aroE,* *aroQ*-II, and *aroB*, followed by distinct DAHP synthase isoenzymes. SA1 and SA2 harbor aroF isoforms (*aroF*-I and *aroF*-II, respectively), SA3 contains *aroH*, and SA4 incorporates a feedback-resistant *aroG* variant (*aroG*^D146N^) from *E. coli*. All constructs were assembled using pSEVA238 as the plasmid backbone (Km^R^, pBBR1 ori, *xylS-Pm* expression system) (A). 3-dehydroshikimic acid (DHS) and shikimic acid (SA) production of strains EM42, EM/SA4 and EMΔXAF/SA4 cultivated in minimal medium supplemented with 20 mM glucose. Metabolite concentrations were determined from samples collected at 72 h of cultivation (B). Data represent mean values ± standard error of the mean (SEM) from at least three biological replicates (n ≥ 3). Statistical significance was assessed using one-way ANOVA followed by Tukey’s post hoc test, with p < 0.05 considered statistically significant

Sequential metabolic engineering of the pathway modules and host background was performed to progressively redirect carbon flux toward SA biosynthesis. Initially, to evaluate whether reinforcing the downstream shikimate pathway could independently drive SA accumulation in *P. putida*, four distinct expression modules (SA1–SA4) were engineered using Gibson Assembly [50] in the backbone pSEVA238. Assembly yielded plasmids SA1, SA2, SA3, and SA4, each carrying a multi-gene synthetic operon under the control of the strong *Pm* promoter (activated by XylS/3-methylbenzoate). Each module was designed to overexpress a core set of endogenous enzymes: *aroB* (3-dehydroquinate synthase), *aroQ-II* (3-dehydroquinate dehydratase), and *aroE* (shikimate dehydrogenase) from *P. putida*. To evaluate the specific contribution of the initial committed step to pathway throughput, each module was integrated with a different DAHP synthase isoenzyme. Specifically, modules SA1, SA2, and SA3 employed the native isoenzymes *aroF-I*, *aroF-II*, and *aroH*, respectively, while module SA4 employed a heterologous feedback-resistant variant from *E. coli, aroG*^D146N^, selected to relieve allosteric inhibition by aromatic end-products and to enhance carbon flux toward aromatic metabolite biosynthesis (Fig. 1A). The gene order in these operons was arranged “downstream-first” to prioritize expression of later pathway steps [30]. This modular approach enabled a comparative evaluation of each isoenzyme's specific impact on overall pathway flux and the accumulation of intermediate metabolites DHS and SA. The modules were initially constructed in *E. coli* CC118, yielding the recombinant strains CC118/SA1, CC118/SA2, CC118/SA3, and CC118/SA4 (Table S1). Subsequently, they were transferred into *P. putida* EM42 via conjugation to evaluate shikimic acid biosynthesis. The resulting engineered strains were designated as EM/SA1, EM/SA2, EM/SA3, and EM/SA4, respectively (Table S1). To assess the SA production capacity of the engineered strains, shake-flask experiments were conducted. Expression of the SA modules, under the control of the *Pm* promoter, was induced with 1 mM 3-methylbenzoate (3-MB) when the cultures reached an OD_600_ of 0.5. Although batch cultures were systematically monitored at 24, 48, and 72 h of cultivation, SA levels remained below the limit of detection during the early stages. Quantifiable titers only emerged for the SA4 module after 72 h of incubation (Fig. 1B). In contrast, the SA1–SA3 modules failed to exhibit significant improvements in SA titers compared to the parental control throughout the 72 h period (data not shown).

The accumulation of 13 mg L⁻^1^ DHS alongside a baseline SA titer of 0.25 mg L⁻^1^ suggests that carbon flux favors DHS formation over SA in strain EM42. The integration of the SA4 module into EM42 (EM/SA4) partially alleviated this imbalance, increasing SA production to 0.7 mg L⁻^1^ while simultaneously reducing the DHS pool to 10 mg L⁻^1^ (Fig. 1B). To minimize the metabolic diversion of phosphoenolpyruvate (PEP) toward pyruvate and maximize its availability for the shikimate pathway [42, 78], the engineered chassis strain ΔXAF (unpublished data) was employed. Deletion of *hexR* was intended to de-repress glucose catabolic genes, thereby enhancing both erythrose-4-phosphate (E4P) and NADPH fluxes [36, 79]. The SA4 module was subsequently integrated into the ΔXAF chassis, yielding EMΔXAF/SA4. Although SA titers did not differ significantly among engineered strains (p > 0.05), a progressive reduction in the DHS pool (13 → 10 → 8 mg L⁻^1^) was observed across successive engineering steps, suggesting improved carbon flux toward SA (Fig. 1B). The persistent DHS accumulation, despite *aroE* overexpression, suggests that the reduction in the DHS pool does not translate proportionally into increased SA titers, indicating that precursor supply may be the dominant limiting factor in these strains.

### Enhancing precursor availability as a key driver for shikimate production in *P. putida*

**Fig. 2:**
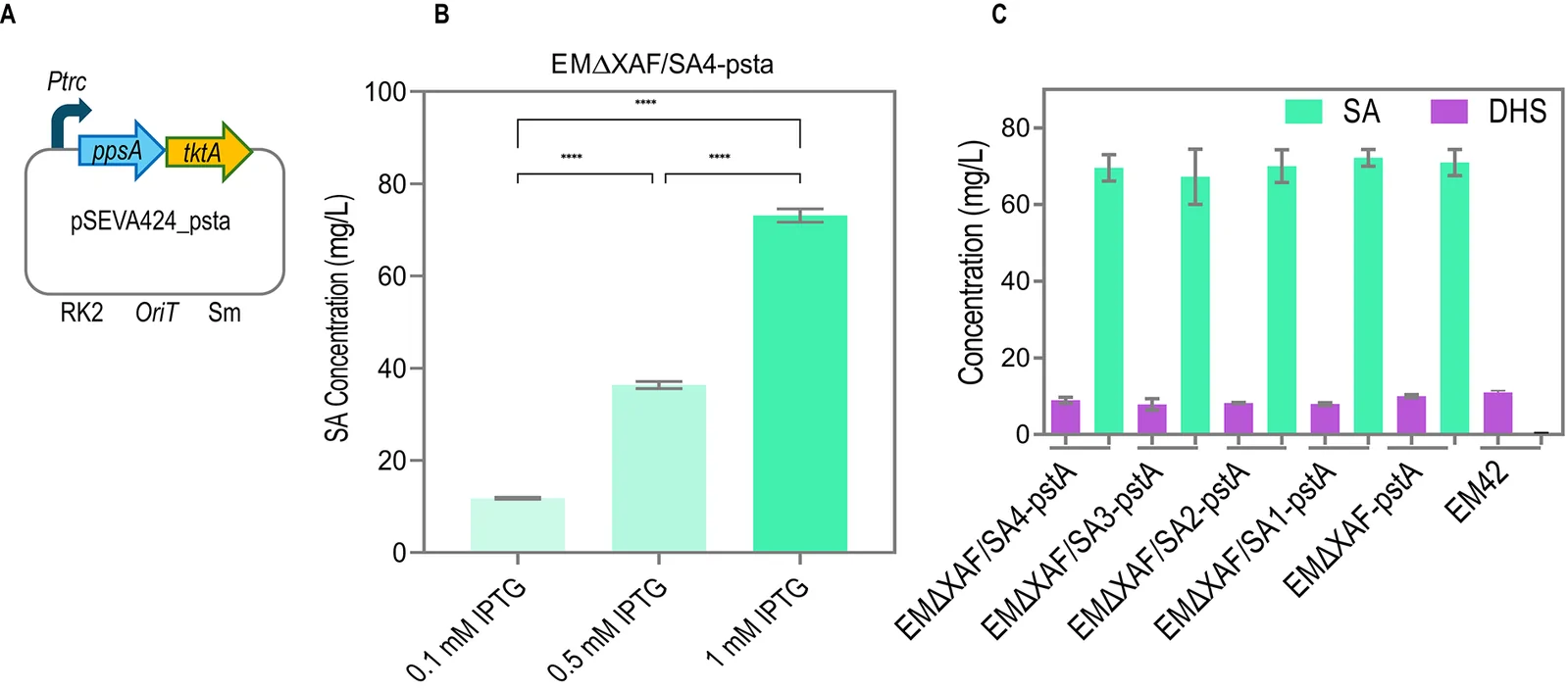
DHS and SA production in engineered *P. putida* strains. Shikimic acid (SA) production by the engineered strain EMΔXAF/SA4-psta harboring the pSEVA424-psta construct under different IPTG induction levels (0.5-1 mM) in the presence of 1 mM 3-methylbenzoate (3-MB). pSEVA424-psta (not to scale, Table S1) (A). The highest SA titer was observed at 1 mM IPTG combined with 1 mM 3-MB (B). Shikimic acid (SA) and dehydroshikimic acid (DHS) titers at 72 h in *P. putida* strains harboring distinct SA biosynthesis modules (SA1–SA4) in the EMΔXAF-psta background, induced with IPTG and 3-MB. The parental strain EMΔXAF-psta and the wild-type EM42 are included as references (C). Data represent mean values ± standard error of the mean (SEM) from at least three biological replicates (n ≥ 3). Statistical significance was assessed using one-way ANOVA followed by Tukey’s post hoc test, with p < 0.05 considered statistically significant

To enhance the availability of intracellular precursors in *P. putida*, a synthetic bicistronic operon was constructed on the pSEVA424 plasmid (Sm^R^, RK2 *ori*, *lacIq*-P_*trc,*_ inducible by IPTG) to enable the coordinated overexpression of *ppsA* and *tktA* (Fig. 2A).Transformation of the genome-edited EMΔXAF/SA4 strain with this plasmid yielded the EMΔXAF/SA4-psta chassis designed to maximize carbon flux toward the shikimate pathway. Under induction with 1 mM (3-MB) to express the SA modules together with different concentrations of IPTG (0.1, 0.5 and 1 mM), EMΔXAF/SA4-psta exhibited induction-dependent increases in SA titers, reaching a maximum of 71 mg L⁻^1^ at 1 mM IPTG (Fig. 2B). This represents a > 62-fold enhancement in SA production relative to the EMΔXAF/SA4 parental strain, underscoring the critical role of precursor supply (PEP and E4P) in limiting pathway throughput. Figure 2C depicts the subsequent evaluation of alternative DAHP synthase isoforms (modules SA1, SA2, and SA3) in the same genetic background, revealing no statistically significant differences in either DHS (~ 10 mg L⁻^1^) or SA (~ 71 mg L⁻^1^) accumulation at 72 h. This plateau in production suggests that once precursor limitations are alleviated, the system likely reaches a saturation point defined by native metabolic constraints. This outcome suggests that the native chromosomal shikimate pathway enzymes of *P. putida* are not themselves rate-limiting once adequate precursor pools are established; rather, the ceiling in SA accumulation likely reflects downstream native constraints that are not overcome by heterologous enzyme overexpression alone. Notably, assessment of the specific contribution of precursor availability to SA biosynthesis using strain EMΔXAF-psta showed similar performance, accumulating DHS and SA at levels statistically comparable to those observed in EMΔXAF/SA4-psta. These observations indicate that under the tested conditions, enhancing PEP and E4P availability alone is sufficient to drive the native chromosomal shikimate pathway of *P. putida* to its current maximum capacity, yielding titers equivalent to those achieved through the overexpression of the selected pathway genes.

### Growth and glucose consumption profiles of engineered strains

**Fig. 3:**
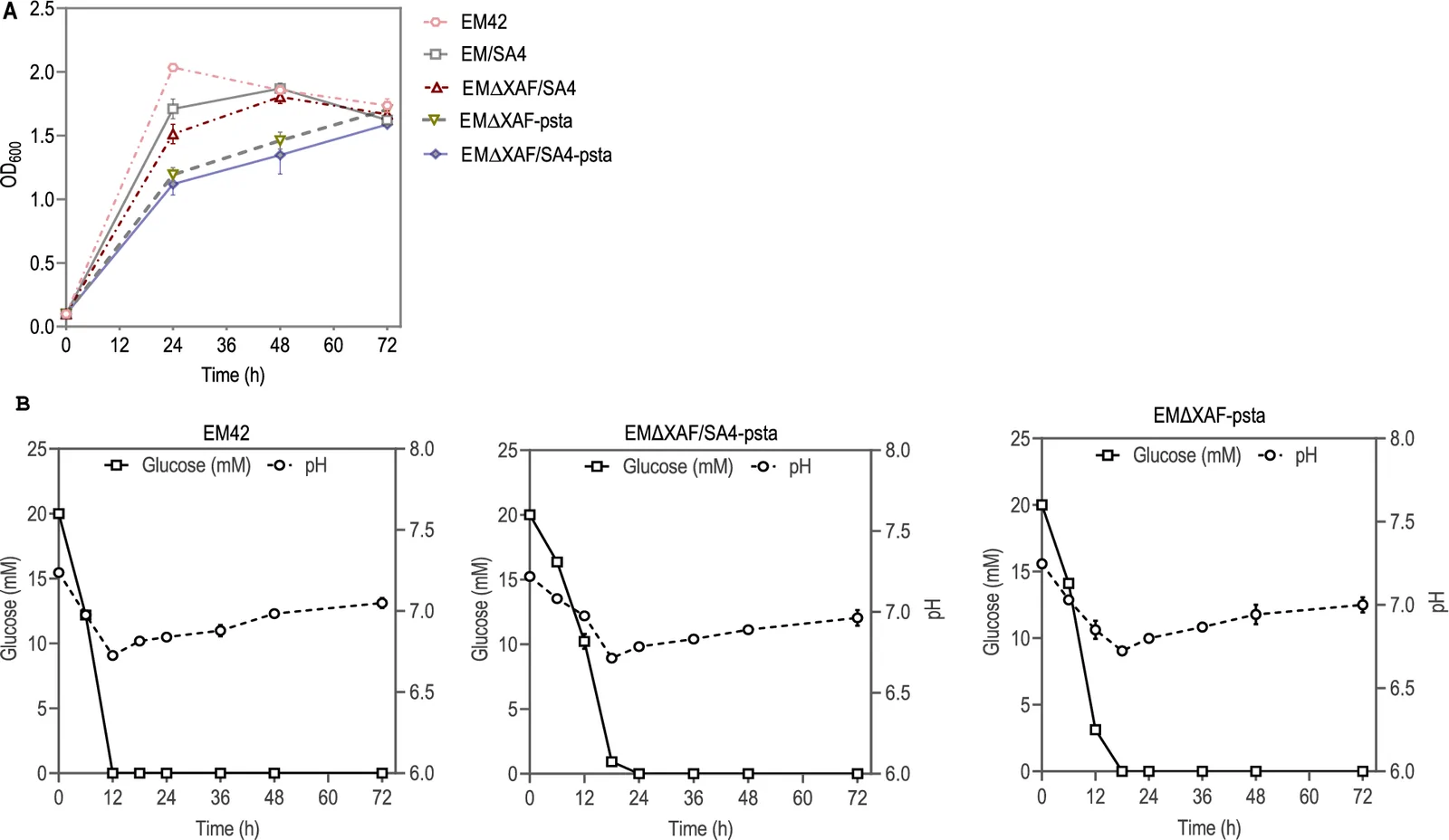
Growth, glucose consumption, and pH profiles of *P. putida* EM42 and its derivative strains. Comparative growth kinetics of engineered *P. putida* strains over 72 h in M9 medium with 20 mM glucose (**A**), Correlation between carbon source depletion and medium acidification for the wild-type (EM42), *ppsA-tktA* overexpressing strains EMΔXAF/SA4-psta and EMΔXAF-psta (**B**). Data represent mean values ± standard error of the mean (SEM) from at least three biological replicates (n ≥ 3). Statistical significance was assessed using one-way ANOVA followed by Tukey’s post hoc test, with p < 0.05 considered statistically significant

The physiological performance of the engineered strains was assessed by monitoring growth dynamics over a 72-h cultivation period (Fig. 3A). The glucose consumption rates were evaluated for the highest SA-producing strains (EMΔXAF-psta and EMΔXAF/SA4-psta) in comparison with the EM42 strain in M9 medium supplemented with 20 mM glucose (Fig. 3B).

Average growth rates were calculated based on optical density measurements obtained during the 0–24 h interval. The parental strain EM42 exhibited an average growth rate of 0.125 h⁻^1^. The engineered strains EM/SA4 and EMΔXAF/SA4 showed growth rates of 0.118 h⁻^1^ and 0.113 h⁻^1^, respectively. Strains carrying *psta*, including EMΔXAF-psta and EMΔXAF/SA4-psta, exhibited growth rates of 0.103 h⁻^1^ and 0.099 h⁻^1^ within the same time period. Across the full cultivation period, all strains continued to grow and reached comparable final cell densities. While EM42 depleted the substrate within 12 h, total exhaustion was delayed until 18 h and 24 h for EMΔXAF-psta and EMΔXAF/SA4-psta, respectively. Despite these different kinetic profiles, all genotypes exhibited highly consistent pH profiles (Fig. 3B), characterized by an initial acidification phase followed by stabilization. This indicates that overall pH homeostasis was maintained in all tested environments.

Notably, despite glucose depletion occurring within the first 24 h of cultivation in both strains (Fig. 3B), extracellular SA concentrations continued to increase progressively throughout the stationary phase, reaching peak titers at 72 h (Supplementary Fig. 1). This post-depletion accumulation could be attributed to the gradual secretion of intracellularly retained SA into the extracellular medium following cessation of active glucose catabolism.

### Transcriptomic analysis of engineered strains

Since no statistically significant difference in DHS or SA titers was observed among the various DAHP synthase isoforms, EMΔXAF/SA4-psta was prioritized for further characterization. This strain carries the SA module along with the overexpression of the feedback-resistant *aroG*^D146N^, a variant frequently prioritized in metabolic engineering studies for its robust catalytic performance [36, 80–85]. To provide a deeper understanding of the underlying metabolic shifts, comparative transcriptomic profiling of EMΔXAF/SA4-psta was performed relative to the wild-type EM42 and EMΔXAF-psta strains.

The transcriptional landscape was evaluated using samples harvested at 72 h from cultures grown in M9 minimal medium supplemented with 20 mM glucose in the presence of 3-MB/IPTG induction. In total, 1253 genes in EMΔXAF-psta and 903 genes in EMΔXAF/SA4-psta were significantly differentially expressed relative to EM42 (padj < 0.05 and |FC|≥ 2) (Table S4). Notably, a high degree of transcriptional congruence was observed, with approximately 70% of these differentially expressed genes (DEGs) exhibiting overlapping expression patterns between the two engineered strains (padj < 0.05 only). For enrichment analyses, a sensitivity analysis was additionally applied using relaxed fold-change thresholds (|FC|≥ 0 for global shifts; |FC|≥ 1 for pronounced responses) to capture broader metabolic trends beyond individual DEGs.

**Fig. 4:**
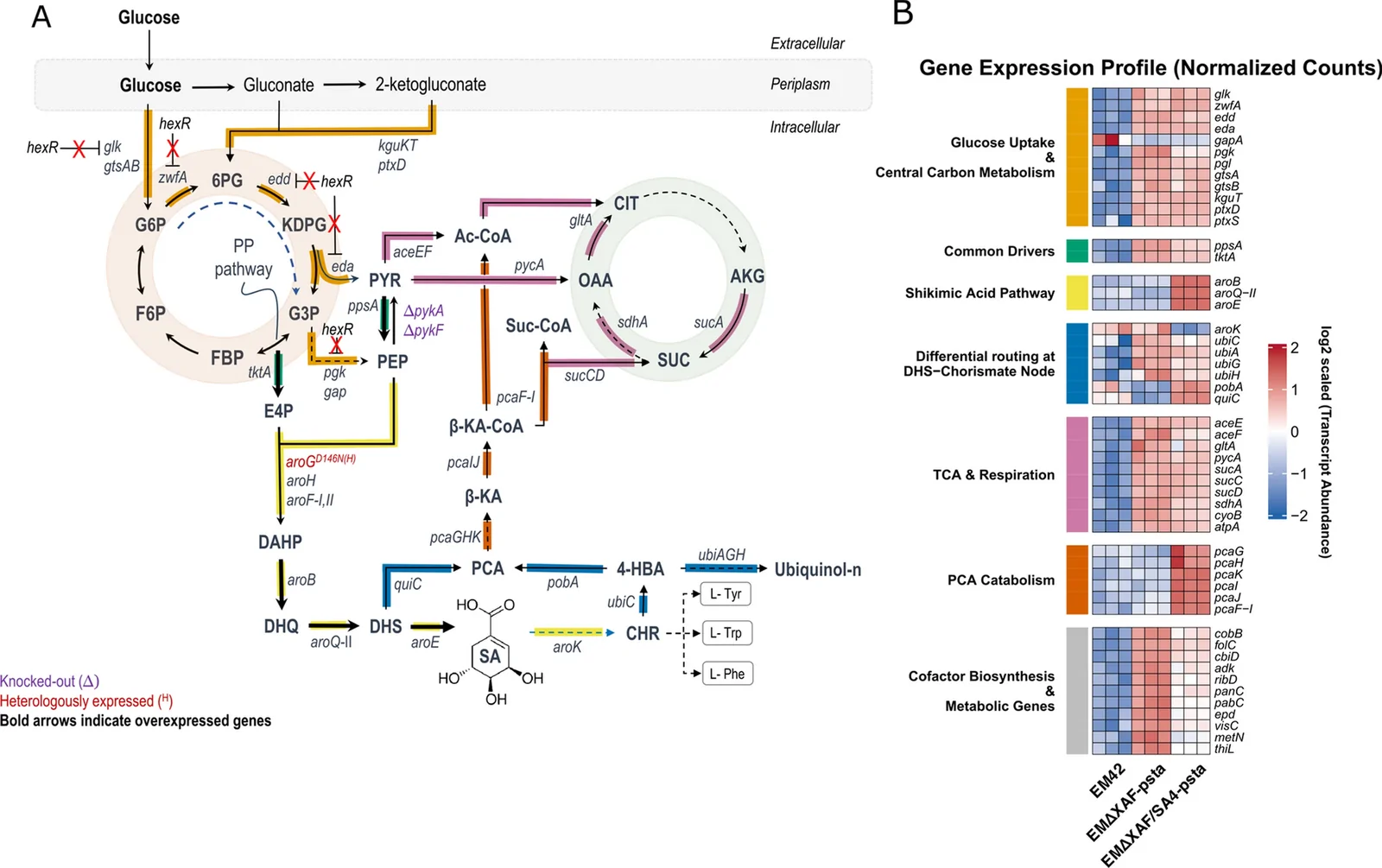
Overview of metabolic engineering strategies and transcriptomic responses in engineered *P. putida* strains. Schematic representation of the engineered central carbon and aromatic biosynthesis pathways. Red crosses indicate gene repression mediated by HexR; genes shown in purple denote chromosomal deletions (Δ*pykA*, Δ*pykF*); bold arrows represent overexpressed native genes; and red superscript (H) indicates heterologously expressed genes. (**A**). Heatmap of log₂ fold changes in transcript abundance for key genes grouped by metabolic module, compared across the parental strain EM42, EMΔXAF-psta, and EMΔXAF/SA4-psta. Color-coded bars on the left denote metabolic modules: gold (glucose uptake and central carbon metabolism), green (common drivers), yellow (shikimate acid pathway), blue (differential routing at DHS–chorismate node), pink (TCA and respiration), orange (PCA catabolism), and gray (cofactor biosynthesis and metabolic genes). Color scale ranges from dark blue (low expression) to red (high expression) (**B**). *2K6PG* 2-keto-6-phosphogluconate, *G6P* glucose-6-phosphate, *6PG* 6-phosphogluconate, *F6P* fructose-6-phosphate, *FBP* fructose-1,6-bisphosphate, *G3P* glyceraldehyde-3-phosphate, *DHAP* dihydroxyacetone phosphate, *PEP* phosphoenolpyruvate, *E4P* erythrose-4-phosphate, *PYR* pyruvate, *AcCoA* acetyl-coenzyme A, *OAA* oxaloacetate, *CIT* citrate, *AKG* alpha-ketoglutarate, *SUC* succinate, *DAHP* 3-deoxy-D-arabino-heptulosonate-7-phosphate, *DHQ* 3-dehydroquinate, *DHS* 3-dehydroshikimate, *SA* shikimate, *CHR* chorismate, *4-HBA* 4-hydroxybenzoic acid, *PCA* protocatechuate, *β-KA* beta-ketoadipate, *β-KA-CoA* beta-ketoadipyl-coenzyme A, *Suc-CoA* succinyl-coenzyme A

The overall metabolic rewiring and pathway-specific gene expression differences between the two strains are summarized in Fig. 4, which integrates a comprehensive metabolic map of all genetic modifications with a heatmap of normalized transcript abundances across seven functionally annotated modules.

### Transcriptomic rewiring of central carbon metabolism

**Fig. 5:**
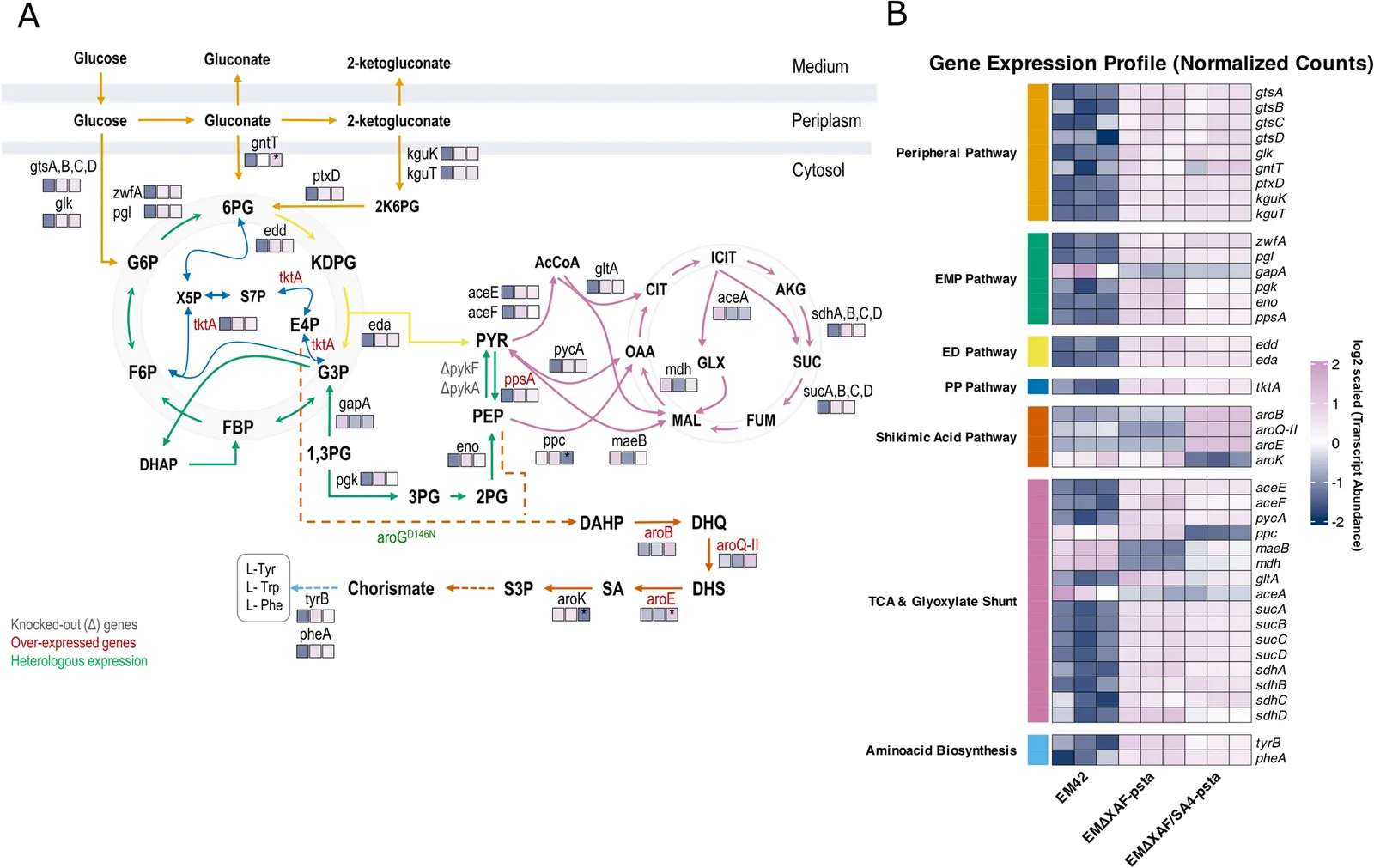
Central carbon metabolic architecture and transcriptomic landscape of engineered *Pseudomonas putida* strains. Schematic overview of the central carbon metabolism in *P. putida*, encompassing the Embden–Meyerhof–Parnas (EMP), Entner–Doudoroff (ED), and Pentose Phosphate (PP) pathways, as well as the TCA cycle and glyoxylate shunt. The diagram traces carbon flux from extracellular glucose uptake through periplasmic oxidative routes and cytosolic catabolism toward aromatic amino acid biosynthesis via the shikimate pathway. Genetic interventions are denoted as follows: gray shading indicates chromosomal knockouts (Δ), red text denotes overexpressed native genes, and green text indicates heterologously expressed genes. Arrow colors correspond to the pathway groupings used in panel B: orange (peripheral uptake), green (EMP), yellow (ED), blue (PP), magenta (TCA and glyoxylate shunt), and orange-dashed (shikimate pathway). Heatmap boxes adjacent to each gene name represent median transcript abundances across biological replicates for EM42, EMΔXAF-psta, and EMΔXAF/SA4-psta (left to right), scaled from dark blue (low expression) to lilac (high expression). Asterisks (*) indicate genes with statistically significant differential expression (adjusted *p* < 0.05, |log₂FC|> 2) in the indicated condition only; genes significant in both strains are displayed without asterisks (**A**). Clustered heatmap of log₂-scaled normalized transcript abundances for genes spanning the peripheral uptake, EMP, ED, PP, shikimate, TCA and glyoxylate shunt, and aromatic amino acid biosynthesis modules. Expression profiles are compared across the parental strain EM42, the precursor-supply-optimized EMΔXAF-psta, and the final SA-producing strain EMΔXAF/SA4-psta (**B**). Metabolite abbreviations: *2K6PG* 2-keto-6-phosphogluconate, *G6P* glucose-6-phosphate, *6PG* 6-phosphogluconate, *2KDPG* 2-keto-3-deoxy-6-phosphogluconate, *F6P* fructose-6-phosphate, *FBP* fructose-16-bisphosphate, *G3P* glyceraldehyde-3-phosphate, *DHAP* dihydroxyacetone phosphate, *13PG* 1,3-bisphosphoglycerate, *3PG* 3-phosphoglycerate, *2PG* 2-phosphoglycerate, *PEP* phosphoenolpyruvate, *X5P* xylulose-5-phosphate, *S7P* sedoheptulose-7-phosphate, *E4P* erythrose-4-phosphate, *PYR* pyruvate, *AcCoA* acetyl-coenzyme A, *OAA* oxaloacetate, *CIT* citrate, *ICIT* isocitrate, *AKG* alpha-ketoglutarate, *SUC* succinate, *FUM* fumarate, *MAL* malate, *GLX* glyoxylate, *DAHP* 3-deoxy-D-arabino-heptulosonate-7-phosphate, *DHQ* 3-dehydroquinate, *DHS* 3-dehydroshikimate, *SA* shikimate, *S3P* shikimate-3-phosphate, *EPSP* 5-enolpyruvylshikimate-3-phosphate

Transcriptomic analysis of the engineered *P. putida* strains EMΔXAF-psta and EMΔXAF/SA4-psta revealed highly consistent expression patterns regarding glucose catabolism, indicating an intensive metabolic effort to enhance glucose import (Fig. 4, Fig. 5, Supplementary Fig. 2). Across both engineered backgrounds, the primary glucose ABC transporter system—comprising *gtsA*, *gtsB*, *gtsC*, and *gtsD*—was significantly upregulated, with fold changes ranging from 1.52 to 3.21. Specifically, *gtsA* exhibited the highest induction within this cluster (3.21/2.98-fold). These changes were accompanied by a consistent ~ 2.07-fold increase in gltR-II transcript levels, a key regulator implicated in carbon assimilation in Pseudomonas species (Table S4). At the pathway level, the "ABC transporters" KEGG category reached statistical significance only in EMΔXAF-psta (p.adj < 0.05), suggesting that the overall transcriptional response toward glucose transport was more pronounced in the absence of the SA4 module (Supplementary Fig. 3).

A defining feature of the metabolic landscape was the intense activation of the peripheral pathway. The genes *kguT* and *ptxD*, responsible for 2-ketogluconate transport and processing, showed the highest induction levels in the dataset, with fold changes exceeding 5.42 and 5.66, respectively. The transcriptional repressor of this pathway, *ptxS*, was also upregulated (2.29/2.17-fold), suggesting a robust but regulated flux through the oxidative route. Given the modest fold changes and marginal statistical support observed in both strains, the transcriptional response of *gntT* was not discussed in detail. The deletion of hexR, the central repressor of the Entner–Doudoroff (ED) and pentose phosphate (PP) pathways, reflects the pronounced derepression of these routes. Accordingly, *zwfA* (3.86/4.42-fold), *pgl* (5.22/4.42-fold), *edd* (2.94/2.52-fold), and *eda* (5.00/4.94-fold) were significantly upregulated. Additionally, *glk* (glucokinase) exhibited increased expression (2.04/2.13-fold), supporting the entry of glucose into the phosphorylated sugar pool (Figs. 4A-B, 5B). These results were further validated via qRT-PCR for EMΔXAF/SA4-psta (Supplementary Fig. 4). To maximize PEP conservation and recycling, the deletion of *pykA/pykF* was coupled with the overexpression of *ppsA*, resulting in a 4.6-fold (EMΔXAF-psta) and 3.5-fold (EMΔXAF/SA4-psta) increase in transcript levels. Simultaneously, the non-oxidative pentose phosphate pathway was reinforced via *tktA* overexpression, which showed an induction of 5.7-fold and 4.8-fold, respectively. Moreover, qRT-PCR analysis confirmed the expression of the precursor-supply genes, with *ppsA* and *tktA* exhibiting 2.62- and 5.10-fold increases in expression, respectively, which remained in close agreement with the transcriptomic trends. Overall, these transcriptional shifts are consistent with successful synchronization of PEP and E4P availability, suggesting increased metabolic flux toward the shikimate pathway (Fig. 5A-B; Supplementary Fig. 4).

Building on the upstream metabolic rewiring described above, we next examined transcriptional changes within the shikimate pathway itself.

### Shikimate pathway

In the EMΔXAF/SA4-psta strain, the presence of the SA4 module triggered a robust transcriptional response; specifically, RNA-seq analysis revealed substantial upregulations of *aroE*, *aroB*, and *aroQ-II* by 8.97-, 6.48-, and 6.33-fold, respectively (Fig. 4, Supplementary Fig. 2C). These findings were further corroborated by qRT-PCR, which showed closely aligned increases of 9.21-, 7.02-, and 6.66-fold for the same targets (Supplementary Fig. 4). In comparison, the EMΔXAF-psta strain displayed markedly lower or even repressed levels for these genes (0.17, 1.14, and − 2.17) (Figs. 4, 5). Despite this nearly ninefold difference in the expression of key rate-limiting steps such as *aroE*, the extracellular titers of 3-dehydroshikimate (DHS) and shikimate (SA) remained statistically indistinguishable between the two strains. Specifically, the concurrent downregulation of *aroK* in the EMΔXAF/SA4-psta strain (− 0.75-fold) is in line with the DHS–SA hydroaromatic equilibrium maintained by AroE reversibility. Ultimately, these results demonstrate that solely increasing the transcript abundance of shikimate pathway modules is insufficient to drive increased metabolite accumulation.

### Differential routing of aromatic intermediates at the DHS–chorismate node

Comparative transcriptomic analysis of EMΔXAF-psta and EMΔXAF/SA4-psta revealed distinct patterns of aromatic intermediate routing around the DHS and chorismate nodes, despite both strains exhibiting comparable extracellular DHS and SA titers (Fig. 4). In EMΔXAF/SA4-psta, transcriptional changes were consistent with channeling of aromatic carbon toward protocatechuate (PCA) metabolism. Expression of *pobA* remained unchanged (padj > 0.05), while genes of the core PCA pathway (*pcaG, pcaH, pcaIJ,* and *pcaF*) and the associated transporter *pcaK* were upregulated, suggesting increased processing of PCA-associated intermediates. Additionally, *quiC* (PP_2554), encoding the 3-dehydroshikimate dehydratase that catalyzes the direct conversion of DHS to protocatechuate, showed a statistically significant but modest increase in EMΔXAF/SA4-psta (+ 0.80 log₂FC, padj < 0.05), captured at the |log₂FC|≥ 0 sensitivity threshold, while it was downregulated in EMΔXAF-psta (− 1.14 log₂FC, padj < 0.05), meeting the |log₂FC|≥ 1 threshold (Fig. 4B). This strain-specific differential expression indicates divergent routing of DHS carbon at this metabolic branch point. In contrast, EMΔXAF-psta displayed a transcriptional profile favoring chorismate utilization toward ubiquinone biosynthesis, as reflected by elevated *ubiC* expression (+ 1.65 log₂FC, padj < 0.05), meeting the |log₂FC|≥ 1 threshold, together with reduced *pobA* expression (− 1.04 log₂FC, padj < 0.05), pointing to increased conversion of chorismate to 4-hydroxybenzoate and downstream ubiquinone intermediates (Fig. 4A-B).

### TCA cycle and global metabolic changes

Transcriptomic profiling revealed quantitative differences between the two strains in the regulation of carbon flux into the tricarboxylic acid (TCA) cycle (Fig. 5, Supplementary Fig. 2B). Genes encoding the pyruvate dehydrogenase complex (*aceE*, *aceF*) were upregulated in both strains, though induction was more pronounced in EMΔXAF-psta (*aceE*: 3.69-fold; *aceF*: 2.89-fold) than in EMΔXAF/SA4-psta (*aceE*: 3.40-fold; *aceF*: 1.94-fold). Downregulation of *mdh* and *aceA* was likewise observed in both backgrounds. EMΔXAF-psta additionally showed higher expression of *sdhA* and *sdhD* (but not *sdhBC*) and *sucABCD*, suggesting selective transcriptional activation of the oxidative core of the TCA cycle (Fig. 4B).

Beyond TCA cycle regulation, transcriptomic profiling revealed both shared and strain-specific signatures in peripheral aromatic metabolism and global metabolic resource allocation. Benzoate uptake and its initial catabolic steps were consistently repressed across both strains: the transcriptional regulator *benR* was strongly downregulated in EMΔXAF-psta (− 3.84) and EMΔXAF/SA4-psta (− 3.29), accompanied by coordinated repression of the benzoate dioxygenase cluster *benA* (− 5.20/ − 4.82), *benB* (− 4.28/ − 4.55), *benC* (− 3.69/ − 4.29), and *benD* (− 3.07/ − 3.87) (Table S4). Similarly, downstream catechol catabolism was attenuated in both backgrounds, with *catA-I* and *catA-II* downregulated in EMΔXAF-psta (− 1.32/ − 3.15) and EMΔXAF/SA4-psta (− 1.93/ − 3.68). The gene-concept network analysis further revealed the specific genes driving the enriched downregulated pathways in both strains (Supplementary Fig. 5).

Strain-specific differences emerged at other aromatic entry points. Phenylalanine and tyrosine catabolism was more severely attenuated in EMΔXAF-psta, where *phhA* and *phhB* were deeply repressed (− 8.28 and − 8.48, respectively), compared to the more moderate repression observed in EMΔXAF/SA4-psta (− 4.85 and − 4.58) (Table S4, Supplementary Fig. 4). Notably, *tyrB* and *pheA*, encoding enzymes at the chorismate branch point toward aromatic amino acid biosynthesis, showed higher upregulation in EMΔXAF-psta (2.32- and 2.30-fold, respectively) than in EMΔXAF/SA4-psta (1.61- and 1.95-fold) (Fig. 4). These strain-specific differences extended beyond aromatic catabolism to broader aspects of cellular metabolism. EMΔXAF-psta displayed elevated transcription of cofactor and vitamin biosynthesis genes such as cobalamin (*cobB*, *cbiD*), riboflavin (*ribD*), thiamine (*thiL*), and pantothenate (*panC*), suggesting increased investment in cofactor availability. In contrast, EMΔXAF/SA4-psta showed enrichment of PCA catabolism genes (*pcaGHIJF*), reflecting the intrinsic aromatic funneling capacity of *Pseudomonas* under conditions of elevated shikimate pathway flux, rather than a global catabolic shift (Fig. 4).

## Discussion

Metabolic engineering strategies have been widely applied in a range of microbial hosts to improve the biosynthesis of shikimate pathway–derived compounds [86]. However, optimization of this pathway in *P. putida* remains challenging due to the highly interconnected and tightly regulated nature of its central metabolism [36, 109]. Established engineering approaches—including precursor supply enhancement, overexpression of key pathway genes, integration of feedback-insensitive DAHP synthase variants, and carbon flux redirection—have enabled production of value-added compounds such as anthranilate, muconate, and polyhydroxybutyrate in this host [36, 82, 87]. The present study systematically evaluates these strategies with respect to their differential effects on the accumulation of two central shikimate pathway intermediates, DHS and SA, which has not previously been examined in *P. putida*.

The SA titer achieved in this study (71 mg L⁻^1^) is lower than those reported for optimized strains in other hosts. Engineered *E. coli* strains have yielded up to 1.21 g L⁻^1^ [88] and *B. licheniformis* has reached 21.8 g L⁻^1^ from glucose [89]. However, those high-titer systems rely on the complete deletion of shikimate kinase genes (*aroK* and/or *aroL*) to block SA consumption, which renders the host auxotrophic for aromatic amino acids and vitamins, necessitating costly medium supplementation. The present work was conducted on strictly defined M9 minimal medium with glucose as the sole carbon source, establishing a non-auxotrophic production baseline with direct industrial relevance. Beyond titer comparisons, *P. putida* offers intrinsic metabolic advantages for SA production. Its constitutively high NADPH/NADP⁺ ratio, arising from ED-pathway-centric glucose catabolism [25, 26], directly supports the AroE-catalyzed reduction of DHS to SA—a step that required dedicated engineering to improve in *C. glutamicum* [90]. The well-established aromatic compound tolerance of *P. putida* [16, 91] also provides a natural buffer against product toxicity that constrains other hosts.

To maximize carbon flux toward the shikimate pathway, a specialized chassis strain, ΔXAF (unpublished), was employed. This strain combines deletion of pyruvate kinase isozymes (*pykA*/*pykF*) to reduce PEP drainage with inactivation of the transcriptional repressor HexR, which de-represses the ED and pentose phosphate (PP) pathways to enhance E4P and NADPH availability [36, 92]. Transcriptomic analysis revealed that both engineered strains display strong upregulation of glucose transport (*gtsABCD*), ED pathway enzymes (*zwfA*, *edd*, *eda*), and the kgu-ptx oxidative funnel [21], in agreement with the expected metabolic rewiring driven by *hexR* deletion [92]. Despite HexR being described as a repressor of *gapA* in KT2440, a consistent downregulation of *gapA* (~ − 0.8-fold) was observed in both strains, in agreement with Bujdoš et al. [93].

Previous studies have shown that standardized overexpression of *tktA* and *ppsA* enhances the intracellular availability of E4P and PEP [29, 43, 45, 88, 94, 95, 110] which are critical determinants of DAHP synthase catalytic performance [96]. Here, standalone overexpression of *ppsA*-*tktA* in the ΔXAF background (EMΔXAF-psta) yielded ~ 284-fold higher SA levels relative to the parental chassis, demonstrating that precursor supply is the primary bottleneck. Our results suggest that overexpression of the selected pathway genes does not improve SA production, regardless of precursor supply status. Rather, the data point to additional, as yet uncharacterized, downstream constraints or native regulatory mechanisms—including the unresolved hydroaromatic equilibrium driven by AroE reversibility and the active aroK-mediated drainage of SA—as the dominant barriers to further titer improvement. Accordingly, SA-producing strains exhibited slower growth rates and reduced glucose uptake, in line with a metabolic state in which carbon flux is redirected from biomass formation toward product synthesis [97, 98].

Although DAHP synthases constitute the entry point of the shikimate pathway and have been well characterized in many organisms [99–102], their functional role in *P. putida* remains incompletely understood [33]. In this host, most previous studies have relied on amplification of heterologous *E. coli* genes [32, 103, 104] thereby providing limited insight into native regulatory control. More recently, genomic integration of the feedback-resistant *aroF-I* gene was shown to enhance phenol production in *P. putida* S12 [104] and a feedback-resistant, L-tyrosine–sensitive DAHP synthase variant was reported to be promising for coumaric acid production in *P. putida* KT2440 [98]; however, under the conditions of enhanced precursor supply, we observed no differences in dehydroshikimate or shikimic acid production upon either overexpression of native DAHP synthase isoforms or heterologous expression of *E. coli aroG*^*D146N*^. Collectively, these findings suggest that DAHP synthase isoform is not a primary determinant of shikimic acid levels in *P. putida* when precursor availability is not limiting.

Importantly, even when two distinct engineering strategies—*ppsA*-*tktA* overexpression alone versus SA4 module integration—yielded comparable extracellular SA titers, the underlying transcriptional profiles differed substantially, suggesting that titer alone may not fully reflect the metabolic state achieved through each approach. Comparative transcriptomic analysis revealed a shared transcriptional core in both engineered strains: coordinated upregulation of ED and PP pathway genes (Fig. 5), induction of *aceE* and *aceF*, repression of *aceA* and *mdh*, and downregulation of *benR*, *benABCD*, *catA*-I, and *catA*-II (Supplementary Fig. 5). The induction of pyruvate dehydrogenase complex genes and suppression of the glyoxylate shunt are consistent with glucose-driven central carbon metabolism and broad carbon catabolite repression in Pseudomonas [21, 92, 111]. Furthermore, the significant enrichment of downregulated pathways across both strains—specifically benzoate degradation and aromatic compound metabolism (Supplementary Fig. 3)—points toward a coordinated tightening of the metabolic network to prioritize primary carbon flux.

Despite this shared core, the strains diverged in several respects. At the level of TCA cycle gene expression, EMΔXAF-psta showed more pronounced induction of *sucABCD* and *sdhAD* compared to EMΔXAF/SA4-psta (Fig. 4); as both strains maintained TCA gene upregulation, this divergence is quantitative rather than qualitative, and its flux-level significance remains to be determined by ^13^C-based metabolic flux analysis. Strain-specific differences were also evident in aromatic carbon routing: in EMΔXAF/SA4-psta, upregulation of *quiC*, *pcaGHIJF*, and *pcaK* suggests engagement of the β-ketoadipate pathway, channeling aromatic intermediates toward succinate and fumarate—consistent with the aromatic funneling capacity of *P. putida* [105, 112]—whereas in EMΔXAF-psta, repression of *quiC* alongside elevated *ubiC* and reduced *pobA* transcript levels points to preferential chorismate routing toward ubiquinol biosynthesis [113] (Fig. 4). The deeper repression of *phhA* and *phhB* in EMΔXAF-psta further indicates more extensive attenuation of aromatic amino acid catabolism in this background. Additionally, elevated transcription of cofactor biosynthesis genes (*cobB*, *cbiD*, *ribD*, *thiL*, *panC*) in EMΔXAF-psta suggests increased transcriptional investment in cofactor availability (Fig. 4). Collectively, these transcriptomic differences indicate that the two strains achieve comparable SA titers through metabolically distinct internal states, and that controlling competing aromatic sinks—particularly the β-ketoadipate pathway—may represent a key target for further titer improvement in *P. putida*.

In many efforts to enhance shikimate production in *E. coli*, the accumulation of dehydroshikimate (DHS) has been identified as a major bottleneck [12]. Accordingly, strategies such as disruption of competing branch pathways, *aroE* overexpression, use of metabolic inhibitors, and optimization of cultivation conditions have been applied to limit DHS formation [12, 106, 107]. Despite partial success, multiple studies indicate that hydroaromatic equilibration remains a dominant driver of DHS accumulation, reflecting the reversibility of *aroE*, which sustains a DHS pool through the back-reaction from shikimate [12, 108]. Even protein engineering approaches targeting *aroE*, while effective in reducing DHS levels, resulted in only modest increases in shikimate titers, indicating that suppression of DHS alone is insufficient and that additional constraints—such as cofactor availability and distributed metabolic flux control—become limiting [12]. Furthermore, while Li et al. [42, 107] proposed that *aroE* attenuation is required to redirect flux toward PCA in *P. putida*, our results demonstrate that robust PCA-directed flux is maintained under enhanced precursor supply without programmed *aroE* downregulation (Fig. 4). Moreover, combined *aroE* overexpression and *aroK* downregulation did not alter the DHS–SA balance in strain EMΔXAF/SA4-psta, highlighting a conserved DHS–SA hydroaromatic equilibrium in *P. putida* similar to that observed in *E. coli*.

The transcriptomic divergence between EMΔXAF-psta and EMΔXAF/SA4-psta reveals distinct, strain-specific bottlenecks that provide a rational framework for future metabolic engineering. In EMΔXAF/SA4-psta, the simultaneous downregulation of *aroK* (-0.75-fold) alongside the robust upregulation of *quiC* (0.8-fold), *pobA* (0.51-fold), and the *pcaGH/IJ* cluster (2.34 to 2.74-fold) points toward a potential diversion of carbon away from shikimate (SA). These transcriptional signatures imply that carbon flux is potentially channeled at both the DHS and 4-HBA nodes toward the β-ketoadipate pathway, though definitive flux-level confirmation awaits ^13^C-MFA.

In contrast, the transcriptomic profile of EMΔXAF-psta—characterized by ubiC induction (1.65-fold) in the absence of significant *pcaGH* or *quiC* expression—is consistent with a preferential carbon routing from chorismate toward ubiquinol biosynthesis rather than SA accumulation. Notably, the persistence of a DHS pool in both strains, despite *aroE* overexpression in EMΔXAF/SA4-psta, is likely a reflection of the thermodynamic reversibility of AroE, sustaining a DHS–SA equilibrium that remains resilient to downstream flux alterations.

These transcriptional signatures imply that enhancing SA yields will require the simultaneous modulation of competing pathways across multiple metabolic nodes, rather than single-target interventions. As these conclusions are currently grounded in transcript-level data, further validation is required to confirm their functional impact on the metabolic landscape. Future efforts will focus on integrating 13C-metabolic flux analysis (^13^*C*-MFA) with quantitative proteomics and intracellular metabolite profiling. Such a multi-omics approach will be essential to verify these metabolic signatures, quantify actual carbon distribution, and establish high-resolution flux maps to guide the rational design of next-generation *P. putida* cell factories for aromatic compound production.

## Conclusion

In this study, a substantial enhancement in SA production was achieved compared to the parental EM42 strain. Based on the results obtained, increased precursor availability was identified as the primary factor driving SA accumulation. In contrast, overexpression of the plasmid-encoded *aroE*, *aroB*, *aroQ-II* and *aroG*^D146N^ genes did not provide an additional contribution to SA production. To the best of our knowledge, this is the first report demonstrating in *P. putida* that standalone overexpression of *ppsA-tktA* can substantially enhance SA titers. Comparative transcriptomic analyses of the EMΔXAF/SA4-psta and EMΔXAF-psta strains indicated that similar extracellular dehydroshikimate and shikimate levels can arise from distinct intracellular metabolic configurations. Our findings further suggest that efficient production of shikimate pathway–derived compounds in *P. putida* will require metabolic engineering strategies that consider hydroaromatic equilibrium in a product-oriented manner. Depending on the target metabolite, selectively biasing this equilibrium may favor the accumulation of different aromatic compounds through targeted, host-specific interventions beyond broadly applied, common engineering approaches. Transcriptional attenuation of *aroK* via CRISPR interference and silencing RNAs, as well as post-translational regulation of AroK using FENIX degradation tags, represent promising future directions to further improve SA titers in the engineered *P. putida* strain.

## Supplementary Information

Supplementary Material 1: Figure S1: Time-course of extracellular shikimic acid (SA) accumulation in EMΔXAF-psta and EMΔXAF/SA4-psta during batch cultivation in M9 minimal medium supplemented with 20 mM glucose as the sole carbon source. Samples were collected at 24, 48, and 72 h. Data represent mean ± SEM of three biological replicates (n = 3).

Supplementary Material 2: Figure S2. Comprehensive transcriptomic profiles of central carbon and aromatic biosynthesis pathways in engineered *Pseudomonas putida* strains. Clustered heatmaps of log₂-scaled normalized transcript abundances for the extended gene sets corresponding to the metabolic modules summarized in Figure 5. Expression profiles are shown across the parental strain EM42, the precursor-supply-optimized EMΔXAF-psta, and the SA4-bearing producer strain EMΔXAF/SA4-psta. Color scale ranges from dark blue (low expression) to lilac (high expression). Peripheral uptake, EMP, ED, and PP pathway genes (A). TCA cycle and glyoxylate shunt genes (B). Shikimate pathway and aromatic amino acid biosynthesis genes (C). Gene identifiers are shown as PP locus tags alongside gene names where assigned. Hierarchical clustering was performed on gene expression profiles across all three strains.

Supplementary Material 3: Figure S3. Comparative KEGG pathway enrichment of EMΔXAF-psta and EMΔXAF/SA4-psta using a stringent cutoff. Enrichment analysis was performed using a fold change cutoff of 1 to isolate highly regulated pathways. Shared upregulation was observed for the Ribosome and TCA cycle pathways across both groups.

Supplementary Material 4: Figure S4: Transcriptional profile of central carbon metabolism and the shikimate pathway in *P. putida* KT2440. The graph illustrates the relative mRNA expression levels of key metabolic genes, as determined by qRT-PCR. Data are expressed as log₂ fold changes relative to the wild-type strain. Positive values indicate upregulation, while negative values represent downregulation. Genes are functionally categorized into the shikimate pathway (*aroE*, *aroB*, *aroQ*-II), precursor sourcing (*ppsA*, *tktA*), alternative glycolytic routes (*zwfA*, *edd*, *eda*, *pgl*), and the TCA cycle and anaplerotic reactions (*aceE*, *gltA*, *mdh*, *maeB*, *gapA*, *ppc*). Error bars represent the standard error of the mean (SEM) of three biological replicates (n = 3). Asterisks indicate statistical significance (p < 0.05) as determined by Student's t-test.

Supplementary Material 5: Figure S5. Gene-Concept Network (cnet) visualization of KEGG enrichment for EMΔXAF-psta and EMΔXAF/SA4-psta. The network visualizes the link between enriched KEGG pathways and their associated genes. Yellow nodes represent pathway terms, with size proportional to the number of related genes. Smaller colored nodes represent individual genes, where the color scale indicates the log2 fold change, with a cutoff of 1 (red indicating downregulation). In EMΔXAF-psta, the network highlights associations with glycine, serine and threonine metabolism, degradation of aromatic compounds, valine, leucine and isoleucine degradation, propanoate metabolism, and microbial metabolism in diverse environments (A). In EMΔXAF/SA4-psta, the network displays linkages to glycine, serine and threonine metabolism, sulfur relay system, propanoate metabolism, valine, leucine and isoleucine degradation, microbial metabolism in diverse environments, and lysine degradation (B).

Supplementary Material 6: Table S1. Bacterial strains and plasmids used in this study.

Supplementary Material 7: Table S2. Primers used in this study.

Supplementary Material 8: Table S3. Primers used for qRT-PCR.

Supplementary Material 9: Table S4. Spreadsheet containing transcriptomic data and DEGs identified in RNA-seq analysis comparing *P. putida* strains EM42, EMΔXAF-psta and EMΔXAF/SA4-psta, including log₂ fold change, p-adjusted values.

## Data Availability

All relevant data are included within this manuscript and its supplementary materials. RNA-seq data have been deposited in NCBI Sequence Read Archive (PRJNA1161809). Raw RNAseq data and the analysis pipeline is published on Zenodo repository (https://doi.org/10.5281/zenodo.18521090).
