# Supplementary material for: Comparative analysis of precursor supply and pathway reinforcement strategies for shikimic acid biosynthesis in Pseudomonas putida
Source: Microb Cell Fact. 2026 Jul 22;25:216. doi: 10.1186/s12934-026-03042-6 (PMC13678936; doi:10.1186/s12934-026-03042-6)
Supplement: Supplementary file 1 [file 12934_2026_3042_MOESM1_ESM.pdf]

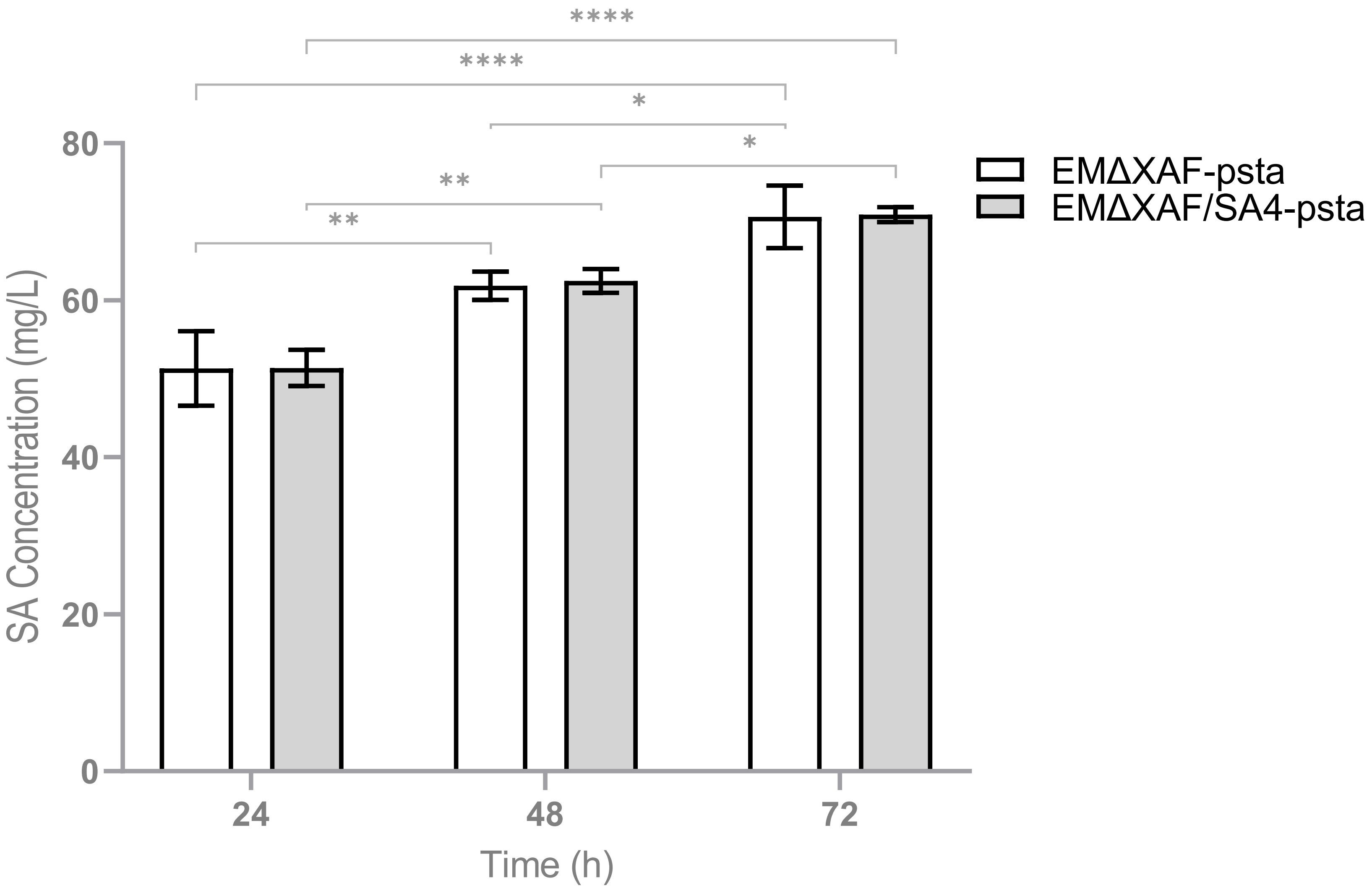

****
****
*
*
**
**
80
60
40
20
0
SA Concentration (mg/L)
24
48
72
Time (h)
EMΔXAF-psta
EMΔXAF/SA4-psta
