# Supplementary material for: Comparative analysis of precursor supply and pathway reinforcement strategies for shikimic acid biosynthesis in Pseudomonas putida
Source: Microb Cell Fact. 2026 Jul 22;25:216. doi: 10.1186/s12934-026-03042-6 (PMC13678936; doi:10.1186/s12934-026-03042-6)
Supplement: Supplementary file 2 [file 12934_2026_3042_MOESM4_ESM.pdf]

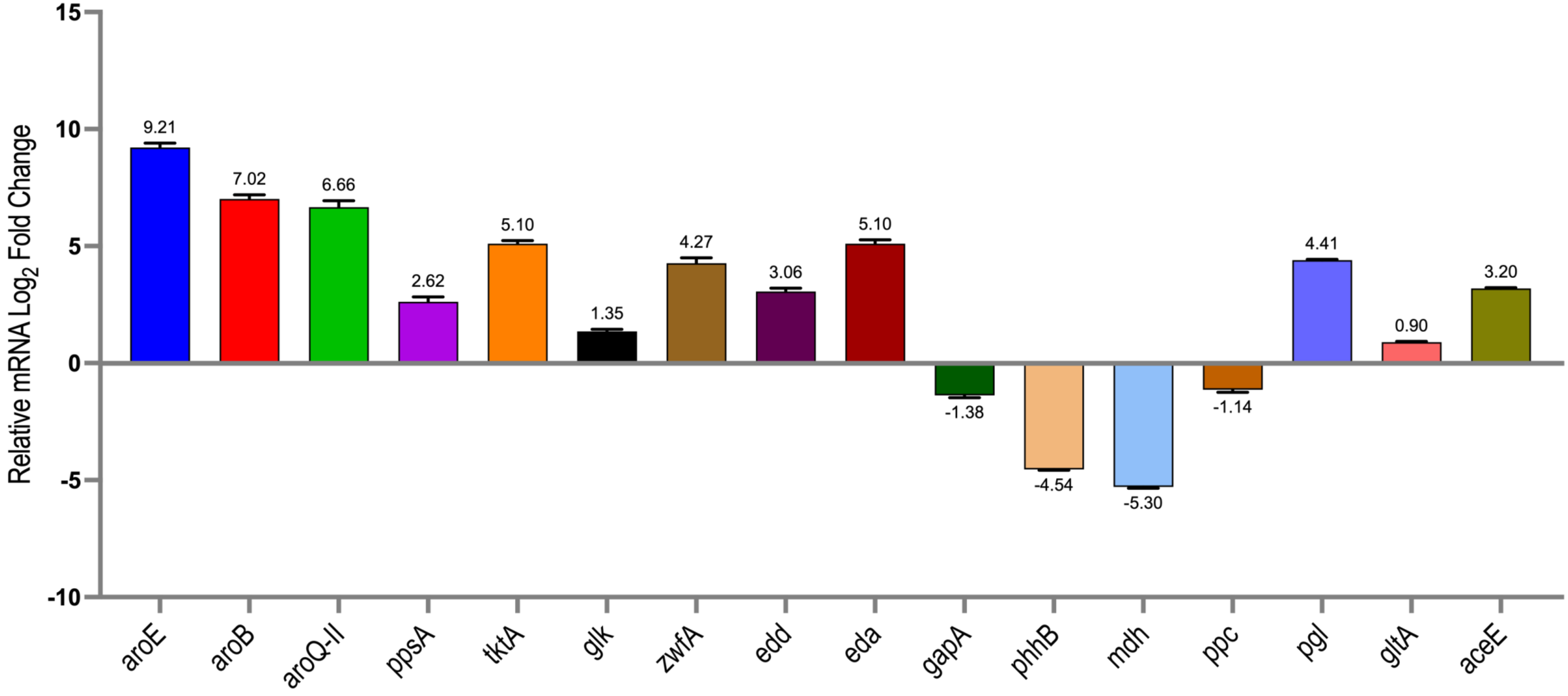

Relative mRNA Log_2 Fold Change
15
10
5
0
-5
-10
9.21
7.02
6.66
2.62
5.10
1.35
4.27
3.06
5.10
-1.38
-4.54
-5.30
-1.14
4.41
0.90
3.20
aroE
aroB
aroQ-II
ppsA
tktA
glk
zwfA
edd
eda
gapA
phhB
mdh
ppc
pgl
gltA
aceE
