# Supplementary material for: Comparative analysis of precursor supply and pathway reinforcement strategies for shikimic acid biosynthesis in Pseudomonas putida
Source: Microb Cell Fact. 2026 Jul 22;25:216. doi: 10.1186/s12934-026-03042-6 (PMC13678936; doi:10.1186/s12934-026-03042-6)
Supplement: Supplementary file 3 [file 12934_2026_3042_MOESM6_ESM.docx]

| **Table S1 Bacterial strains and plasmids used in this study** | | |
| --- | --- | --- |
| **Strain** | **Description** | **References** |
| *E. coli* strains |  |  |
| DH5α | Cloning host; *supE44, ∆lacU169* (Φ80 *lacZ*∆M15), *hsdR17* (rK-mK^+^), *recA1*, *endA*1, *thiE*1, *gyrA*, *relA* | 50 |
| CC118 | Cloning host; Δ(*ara-leu*) *araD* Δ*lacX*74 *galE*, *galK* *phoA thiE1* *rpsE*(Sp^R^), *rpoB*(Rif^R^), *argE*(Am), *recA1* | 53 |
| HB101 | Helper strain used for conjugation; F- , λ- , *hsdS20* (rB- mB- ), *recA*13 *leuB6*(Am), *araC*14 Δ(*gptproA*)62*, lacY1 galK2*(Oc), *xyl-5*, *mtl-1*, *thiE1*, *rpsL*20 ( Sm^R^), *glnX*44 (AS) | 52 |
| CC118/SA1 | The plasmid SA1 was transformed into CC18 | This work |
| CC118/SA2 | The plasmid SA2 was transformed into CC18 | This work |
| CC118/SA3 | The plasmid SA3 was transformed into CC18 | This work |
| CC118/SA4 | The plasmid SA4 was transformed into CC18 | This work |
| ***P. putida* strains** |  |  |
| KT2440 | mt-2 derivative cured of TOL plasmid pWW0 | 51 |
| EM42 | KT2440 derivative; Δprophage1 Δprophage4 Δprophage3 Δprophage2 ΔTn7 Δ*endA-1* Δ*endA-2* Δ*hsdRMS* Δflagellum ΔTn*4652* | 47,48,49 |
| EM/SA1 | The plasmid SA1 was transformed into EM42 | This work |
| EM/SA2 | The plasmid SA2 was transformed into EM42 | This work |
| EM/SA3 | The plasmid SA3 was transformed into EM42 | This work |
| EM/SA4 | The plasmid SA4 was transformed into EM42 | This work |
| EMΔX | EM42Δ*hexR* | This study |
| ΔXAF | EM42Δ*hexR*Δ*pykA*Δ*pykF* | Unpublished |
| EMΔXAF/SA1 | ΔXAF harboring plasmid SA1 | This work |
| EMΔXAF/SA2 | ΔXAF harboring plasmid SA2 | This work |
| EMΔXAF/SA3 | ΔXAF harboring plasmid SA3 | This work |
| EMΔXAF/SA4 | ΔXAF harboring plasmid SA4 | This work |
| EMΔXAF/SA4-psta | ΔXAF harboring plasmid SA4 and pSEVA424_psta | This work |
| EMΔXAF-psta | ΔXAF harboring plasmid pSEVA424_psta | This work |
| **Plasmids** |  |  |
| pRK600 | Helper plasmid used for conjugation; *oriV* (ColE1), RK2(*mob*^+^ *tra*^+^); Cm^R^ | 54 |
| pSEVA658-ssr | *xylS-Pm* → ssr, oriV RSF1010; GmR | 56 |
| pSEVA421-Cas9tr | Cas9 and tracrRNA; oriV RK2; SmR/SpR | 56 |
| pSEVA2316 | SEVA CRISPR array; oriV pBBR1; KmR | 57 |
| pSEVA2316-hexR | pSEVA2316 derivative bearing the hexR spacer | This work |
| pSEVA238 | Km^R^, pBBRI, *oriT*; standard broad-host-range plasmid for Gram-negative bacteria | 55 |
| pSEVA424 | Sm^R^, *ori* RK2, *oriT*; standard broad-host-range plasmid for Gram-negative bacteria | 55 |
| pSEVA424_psta | Sm^R^, *ori* RK2, *oriT*; pSEVA424-derivative with *pppsA-tktA* (*psta*) *fragment* (4.3 kb) cloned as a *Xba*I*/Spe*I fragment | This work |
| SA1 | Km^R^, pBBRI, *oriT*; Km^R^, pSEVA238-derivative with *aroE* (825 bp),aroQ-II (450 bp), *aroB* (~1.1 kb), *aroF-I* (~1.1 kb) fragments | This work |
| SA2 | Km^R^, pBBRI, *oriT*; pSEVA238-derivative with *aroE* (825 bp), *aroQ-II* (450 bp), *aroB* (~1.1 kb), *aroF-II* (~1.1 kb) fragments | This work |
| SA3 | Km^R^, pBBRI, *oriT*; pSEVA238-derivative with *aroE* (825 bp), *aroQ-II* (450 bp), *aroB* (~1.1 kb), *aroH* (~1.3 kb) fragments | This work |
| SA4 | Km^R^, pBBRI, *oriT*; pSEVA238-derivative with *aroE* (825 bp), *aroQ-II* (450 bp), *aroB* (~1.1 kb) with *aroG*^D146N^ (1053 bp) from pS3 | This work |
| pS3 | Cm^R^, pBBR1; *lacI* Plac-UV5::*aroE-aroD-aroB*^op^-*aroG*-ppsA-tktA* | Addgene, H505596 |
| * Superscript ‘‘R’’ indicates resistance to the following antibiotics: Km kanamycin, Amp ampicillin, Cm chloramphenicol. | | |
