# Supplementary material for: Comparative analysis of precursor supply and pathway reinforcement strategies for shikimic acid biosynthesis in Pseudomonas putida
Source: Microb Cell Fact. 2026 Jul 22;25:216. doi: 10.1186/s12934-026-03042-6 (PMC13678936; doi:10.1186/s12934-026-03042-6)
Supplement: Supplementary file 4 [file 12934_2026_3042_MOESM8_ESM.docx]

**Table S3.** Primers used for qRT-PCR

| Genes | Forward Primer | Reverse Primer | Product Size |
| --- | --- | --- | --- |
| *aroE* | ACCGGTCAGGACCTGGAATA | AAGGCCTCTTCCTTGAACGG | 125 bp |
| *aroB* | GATCTTCGATGGCCTGCTGA | ACCTGGGACAACAGGGTAGT | 156 bp |
| *aroQ-II* | GCCCCTTATTCTCGTGCTCA | TCACCTTCGTGGTTGGTCTG | 165 bp |
| *ppsA* | GATAAGCTCGGCGTCCATGA | CTGCTCGAGAAAGTCACGGT | 147 bp |
| *tktA* | GGCGAGCCTGAACTGATTCT | CTTGTACCAGAAGTCGGCGT | 222 bp |
| *zwfA* | AAGAGACGGTGCAGAACCTG | CTGCAGCAGGTGATTCTGGA | 185 bp |
| *edd* | AGGCCGACATCAACCACTTC | CACACCAGCTTGCCATTGTC | 160 bp |
| *eda* | CTCAAGGCCATTCAGGTGCT | GCGGAATTTCACTGTCCACG | 172 bp |
| *mdh* | GCTGTGGATTCAGGTTCCGA | CTGTCGAGGATTGGCAGGT | 155 bp |
| *phhB* | GCCTTGAACCAAGCCCATTG | TGACCTTGCCCCATTCAGTC | 247 bp |
| *glk* | GGCGAGTGGCGGGTAATAA | GCCACTCATACACCCCTTGT | 143 bp |
| *rpoD* | AGGAAGGCAACATCGGTCTG | GATCATGTGCACCGGAATGC | 155 bp |
| *gapA* | TACAACGCTCTGCCATTGGT | GAACCCCCACTCGTTGTCAT | 132 bp |
| *ppc* | CAGGACCATCGTGACCTGAC | TGCCACAGTGAATGCTCGAT | 164 bp |
| *pgl* | TCACCTGTTGAAGGGCGAAG | AAGGCCTGGTCAGCTTTGAG | 99 bp |
| *gltA* | CACCGTGATGAAGCAGACCT | GAACATGCTGGTCGGAATGC | 196 bp |
| *aceE* | TCATCGACGGCGAATACCAG | CCACGGTTGAGCTTCCAGAT | 133 bp |
